# Case report: Navigating the challenges: successful mechanical thrombectomy in a case of persistent primitive hypoglossal artery

**DOI:** 10.3389/fneur.2023.1248506

**Published:** 2023-11-24

**Authors:** Kamran Ahmed Khan, Irfan Amjad Lutfi, Jehangir Ali Shah, Salman Farooq, Shahnoor Ahmed, Bashir Ahmed Solangi, Ayan Ahmed Khan, Fazal Zaidi, Ali Ammar, Nadeem Qamar

**Affiliations:** ^1^Department of Adult Cardiology & Neurology, National Institute of Cardiovascular Diseases (NICVD), Karachi, Pakistan; ^2^ Department of Medicine, Aga Khan University Hospital, Karachi, Pakistan; ^3^Student of Foundation Public School, Karachi, Pakistan; ^4^ Department of Neurology, University of Toledo, Toledo, OH, United States

**Keywords:** persistent primitive hypoglossal artery, mechanical thrombectomy, stroke, MCA, PPHA

## Abstract

Persistent primitive hypoglossal artery (PPHA) is a highly uncommon abnormal connection between the internal carotid artery (ICA) and vertebral artery (VA), with reported incidences ranging from 0.027 to 0.26%. Attempting endovascular intervention in such cases presents a considerable challenge as it carries a higher risk of embolization and other procedure-related complications that may affect a wide area of the brain. We present a case study involving the utilization of mechanical thrombectomy (MT) to treat an ischemic stroke in the M1 segment of the middle cerebral artery (MCA) despite the presence of PPHA. Performing mechanical thrombectomy in an anomalous vascular connection is feasible; however, it necessitates heightened vigilance, thorough knowledge of the anatomy, and utmost caution.

## Background

During fetal circulation, an intricate network of anastomoses is established between the carotid and basilar arteries, involving the hypoglossal, trigeminal, optic, and proatlantal arteries. In the normal course of embryogenesis, carotid–vertebro-basilar anastomoses typically regress and cease to exist (1). However, in rare instances, their persistence can occur, representing a carotid–basilar anastomosis that endures beyond the usual developmental period. Persistent primitive hypoglossal artery (PPHA) is an example of these developmental connections between the carotid and vertebro-basilar arteries. The reported prevalence of this anomalous artery in angiographic studies ranges from 0.03 to 0.09% (2). This unique artery is considered a vascular anomaly, which originates from the internal carotid artery (ICA) between the C1 and C2 vertebral levels and then passes through the hypoglossal canal and joins the vertebro-basilar system, creating an anastomosis (3). PPHA can be a potential route for emboli originating from the heart or proximal internal carotid artery (ICA) to reach the posterior circulation, resulting in ischemic stroke (4). Moreover, in patients with PPHA, there is a rare occurrence of simultaneous embolic infarcts in both the anterior and posterior circulation. Additionally, a PPHA and carotid artery dissection can be associated with recurrent cerebral infarction in both the anterior and posterior circulation (5, 6).

## Case presentation

We are reporting a case of a 43-year-old lady healthcare worker with a modified Rankin score (mRS) of zero at baseline, presented to the National Institute of Cardiovascular Diseases (NICVD), Karachi, Pakistan, with right-sided hemiparesis, aphasia, and slightly altered level of consciousness of 3-h duration. Her physical examination revealed right-sided facial drooping and left-sided gaze preference with an overall NIHSS of 18. Her past medical history was positive for ischemic heart disease. Her baseline hemodynamics were within normal limits with a blood pressure (BP) of 131/63 mm Hg.

## Investigations

**Figure 1:**
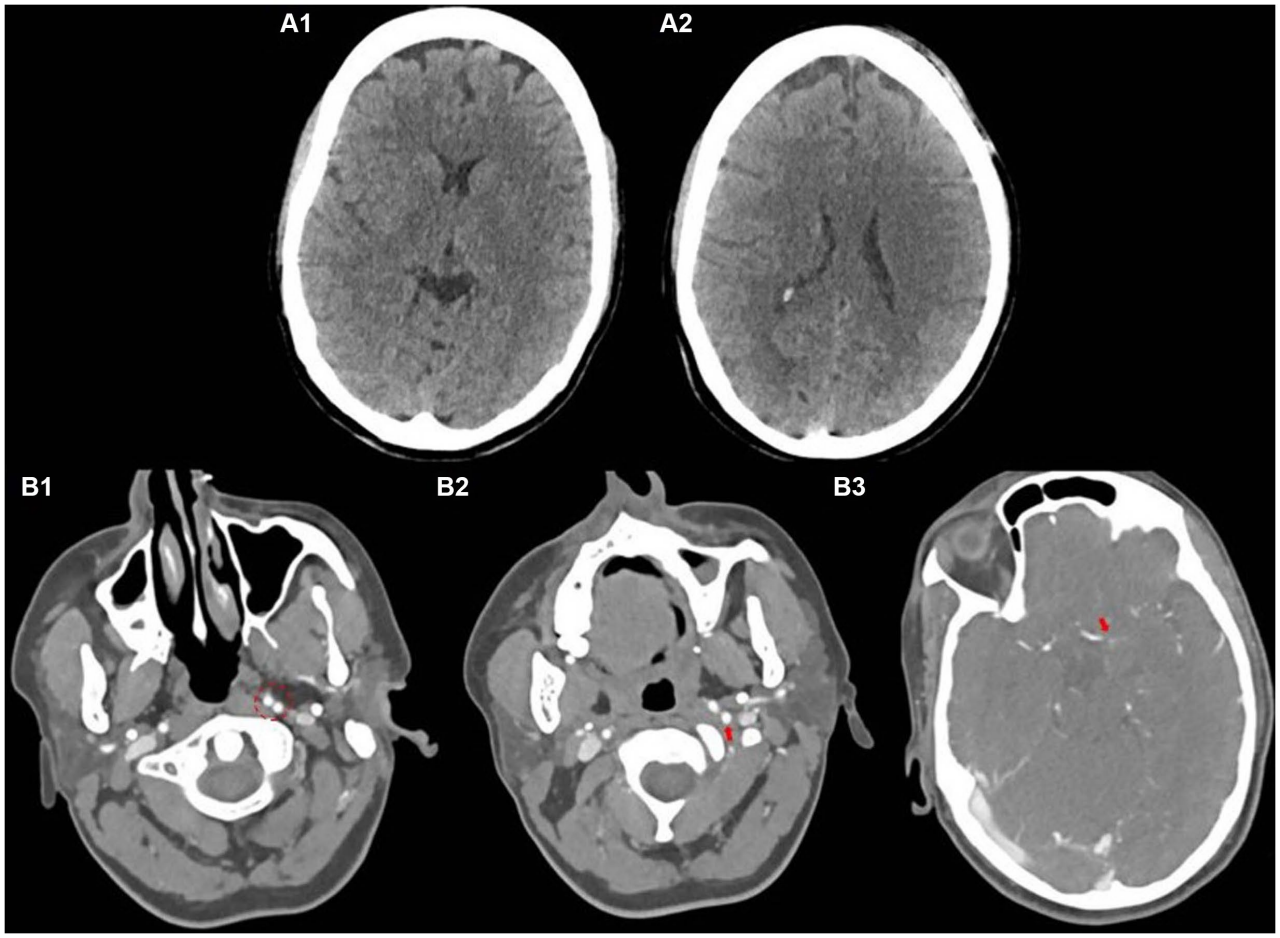
Baseline non-contrast CT scan **(A)**, CT angiography showing bifurcating ICA **(B1)**, persistent primitive hypoglossal artery **(B2)**, and occluded left MCA **(B3)**.

With the provisional diagnosis of stroke, the hyper-acute stroke team was activated and the patient was shifted for computed tomography angiography (CTA) within 15 min of her presentation. Her non-enhanced CT scan is shown in Figures 1A1, 2.

**Figure 2:**
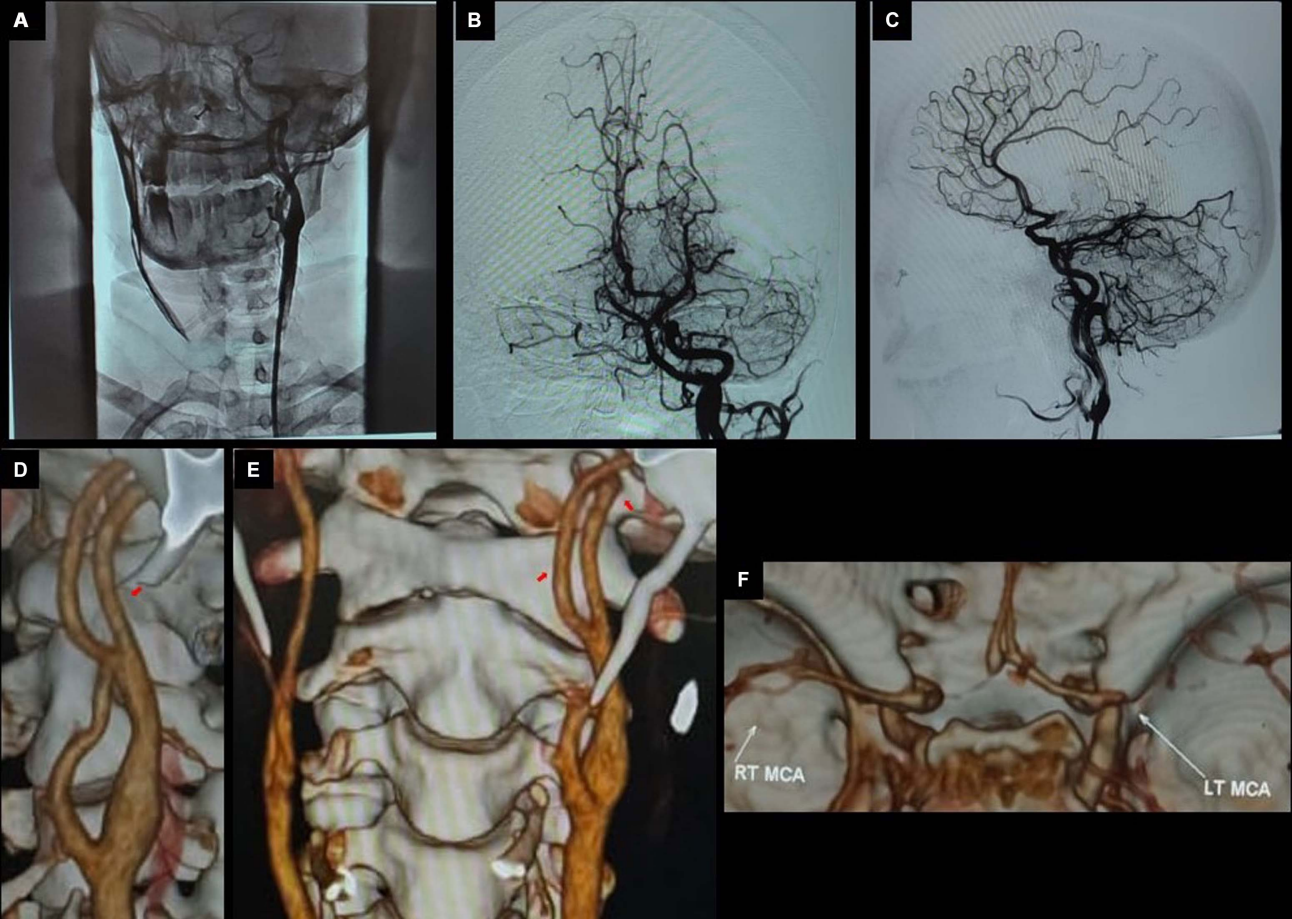
Pre-thrombectomy; LAO view at CCA bifurcation **(A)**, town’s view **(B)**, lateral view **(C)**, and 3-D images showing bifurcating ICA **(D)**, persistent primitive hypoglossal artery **(E)**, and occluded left MCA **(F)**.

Her subsequent CTA (Figures 1B1–3) revealed an occluded left-sided middle cerebral artery (MCA) at the M1 segment. However, an interesting and uncommon occurrence was the bifurcating left internal carotid artery (ICA) at the level of C2, after the common carotid artery (CCA) bifurcation on axial images (Figures 1B1–3) and 3D images as shown in Figure 2, respectively. The actual ICA was present medially, while laterally, it was PPHA, arising from the cervical part of the ICA and continuing as the left vertebral artery connecting the anterior and posterior circulation. PPHA is a fetal connection between anterior and posterior circulation, which failed to regress as expected in the normal developmental process. However, she fulfilled the mechanical thrombectomy (MT) criteria as per American Stroke Association Guidelines 2018 (7) and subsequently shifted to the catheterization laboratory for direct MT without bridging with thrombolytic therapy.

**Figure 3:**
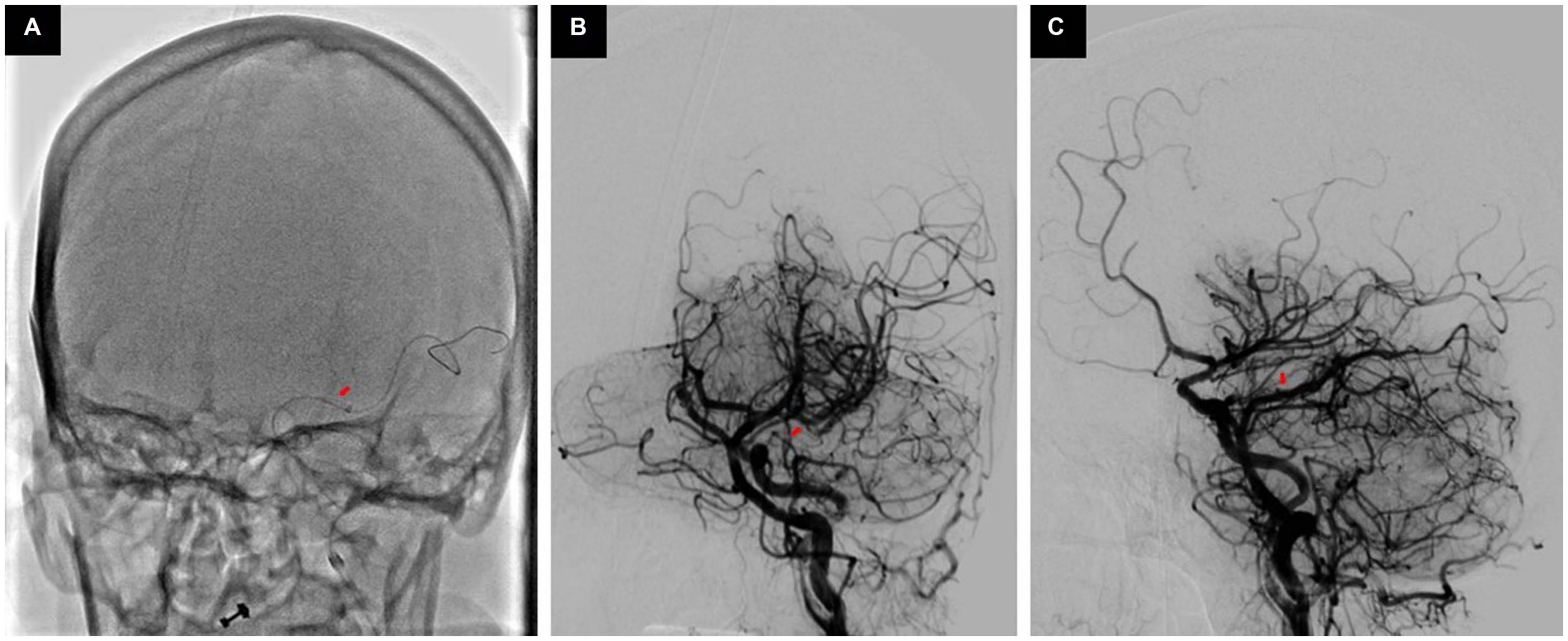
ADAPT is being performed **(A)**, Post-thrombectomy town’s view **(B)** and lateral view **(C)** showing recanalized MCA.

Her baseline digital subtraction angiography (DSA) further confirmed the anomalous connection of left ICA with the left vertebral artery (VA) as PPHA, which resulted in the filling of the entire posterior circulation through left ICA injection, along with an occluded M1 segment of left as shown in Figure 3.

## Treatment

We performed her thrombectomy very cautiously to minimize the risk of embolization in the posterior circulation and avoid unnecessary injection. We performed a direct first pass aspiration technique (ADAPT) only once, which resulted in the successful recanalization of MCA; however, there was some embolization to an A3 segment, as shown in Figure 3.

## Outcome and follow-up

**Figure 4:**
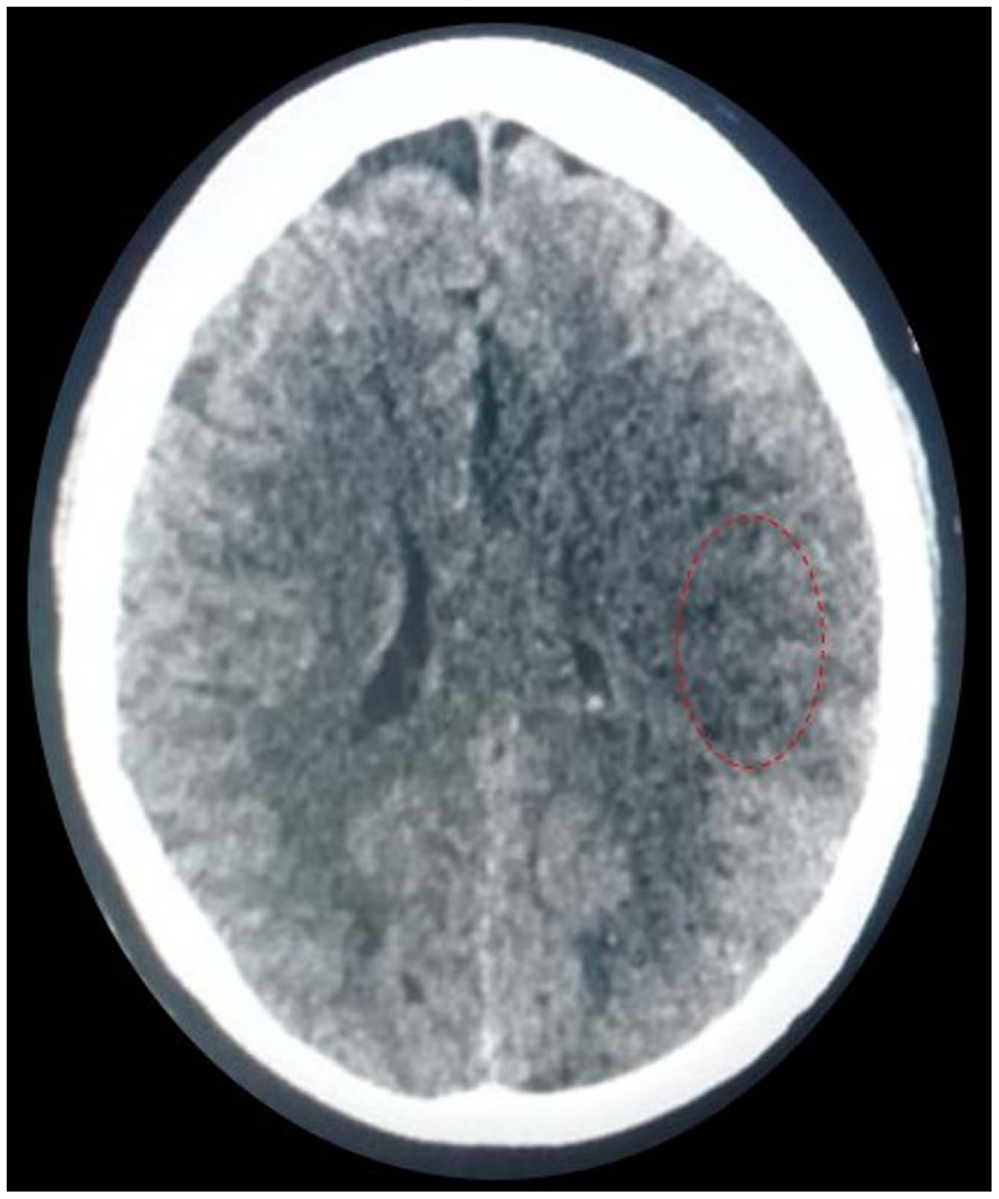
Post-thrombectomy; non-contrast CT scan showing subtle hypodensity in left MCA territory with an ASPECT of 9.

Post-MT, her BP remained stable and below 140 mmHg, which is well within target as mentioned in our institutional protocol post-MT with TICI IIb flow. She was then shifted to our stroke unit, where she was monitored and managed in accordance with standard clinical practice guidelines. Her post-MT CT scan after 24 h showed no hemorrhage; however, she developed subtle hypodensity with an ASPECT of 9 (Figure 4). She was kept in the hospital and treated with IV mannitol along with a single antiplatelet agent and other symptomatic treatment. She was discharged home after 4 days with an mRS of 4. She was subsequently followed in the outpatient department, and after 3 months of follow-up, her mRS improved to 3.

## Consent

We have obtained written consent from the patient’s attendant for reporting this case report.

## Discussion

The PPHA belongs to a category of carotid–basilar anastomoses, which also encompasses the persistent trigeminal, otic, and proatlantal intersegmental arteries. These arteries are designated based on their respective cranial nerve associations. PPHA is a unique and infrequent vascular condition representing an embryonic connection between the ICA and the vertebro-basilar artery system. Specifically, the hypoglossal artery originates from the ICA, typically between the C1 and C2 vertebral levels, and courses through the hypoglossal canal to establish a connection with the vertebro-basilar arterial network. It manifests as a consequence of the incomplete regression of pre-segmental arteries responsible for nourishing the posterior circulation during the early stages of fetal brain development (8). Notably, the left PPHA is more prevalent, accounting for approximately 65% of cases (9).

PPHA ranks as the second most prevalent persistent carotid–vertebro-basilar anastomosis, with an estimated incidence ranging from 0.027 to 0.26%, as mentioned above (10). It exerts substantial hemodynamic alterations on both the carotid and vertebro-basilar vascular systems, potentially correlating with the presence of intracranial vascular anomalies. It typically represents an incidental discovery during angiography, often remaining entirely asymptomatic.

In its presence, the vertebral arteries usually exhibit hypoplasia, the ipsilateral ICA may be entirely absent, and under the circumstances involving occlusion of the contralateral ICA, the PPHA can assume the exclusive role of supplying the circle of Willis. The literature has documented a limited number of cases in which the PPHA serves as the sole source of cerebral blood supply (8). The co-occurrence of intracranial aneurysms alongside PPHA was observed at an incidence rate of approximately 26% (11). An especially noteworthy consequence of PPHA is the occurrence of simultaneous anterior and posterior circulation infarctions in conjunction with carotid atherosclerosis. While this occurrence is infrequent, a limited number of cases have been documented in the existing literature (6).

Only 10–20% of all ischemic strokes qualify for endovascular treatment based on involvement of large vessels and time of presentation since last known to be well (12, 13). There exists a limited timeframe within which this intervention can be successfully performed. As this time elapses, the effectiveness of reperfusion diminishes. Current American Stroke Association Guidelines (ASA) recommend the provision of endovascular treatment up to the window of 6 h, which can be extended up to 24 h since last known to be well with the help of perfusion studies (14).

However, there were no significant differences between the mechanical thrombectomy and control groups regarding rates of symptomatic intracranial hemorrhage or 90-day mortality.

Performing mechanical thrombectomy has always been a complex task due to the varying tortuosity (twisting and turning) of intracranial blood vessels. It requires the use of advanced tools such as guiding catheters, aspiration catheters, micro-catheters, or stent retrievers, often in a multi-step telescoping manner. Moreover, when dealing with anomalous anatomy, the procedure becomes more challenging than in a typical normal setting. However, certain steps, including more selective engagement, use of a balloon-guide catheter, use of concomitant thrombolytic therapy, and if patients presented within 4.5 h, may reduce the risk of embolization in the non-involved territory.

The existence of this abnormal connection poses a significant risk to the overall circulation during the procedure, increasing the likelihood of distal embolization or other mechanical complications.

## Learning points/take home messages

- Performing mechanical thrombectomy in the presence of an anomalous vascular connection is feasible; however, it necessitates heightened vigilance, thorough knowledge of the anatomy, and utmost caution.
- The procedure becomes particularly challenging due to the inherent risks of embolization and other potential complications that can concurrently impact both the anterior and posterior circulation.
- Careful considerations and meticulous execution are essential to minimize the potentially disastrous consequences associated with this unique anatomical condition.

## Data availability statement

The original contributions presented in the study are included in the article/Supplementary material, further inquiries can be directed to the corresponding author.

## Ethics statement

The studies involving human participants were reviewed and approved by the National Institute of Cardiovascular Diseases (NICVD). The patients/participants provided their written informed consent to participate in this study. Written informed consent was obtained from the individual’s legal guardian/next of kin for the publication of any potentially identifiable images or data included in this article.

## Author contributions

KK, IL, JS, and SF conceived the idea of the case report. KK, IL, JS, SF, SA, and BS were involved in the assessment diagnosis and management of the patients. KK, IL, JS, SF, SA, BS, AK, FZ, AA, and NQ were involved in data acquisition, manuscript writing, and critical review of the manuscript. All authors contributed to the article and approved the submitted version.
